# Cardiovascular Development and the Colonizing Cardiac Neural Crest Lineage

**DOI:** 10.1100/tsw.2007.189

**Published:** 2007-07-03

**Authors:** Paige Snider, Michael Olaopa, Anthony B. Firulli, Simon J. Conway

**Affiliations:** Cardiovascular Development Group, Herman B. Wells Center for Pediatric Research, Indiana University School of Medicine, Indianapolis, IN 46202, USA

**Keywords:** cardiac neural crest, migration, pharyngeal arches, Pax3, Wnt1, aortic arch arteries, vascular remodeling, outflow tract, endocardial cushions, cardiac septation, myocardial failure, Cre/loxP, lineage mapping, stem cells

## Abstract

Although it is well established that transgenic manipulation of mammalian neural crest-related gene expression and microsurgical removal of premigratory chicken and *Xenopus* embryonic cardiac neural crest progenitors results in a wide spectrum of both structural and functional congenital heart defects, the actual functional mechanism of the cardiac neural crest cells within the heart is poorly understood. Neural crest cell migration and appropriate colonization of the pharyngeal arches and outflow tract septum is thought to be highly dependent on genes that regulate cell-autonomous polarized movement (i.e., gap junctions, cadherins, and noncanonical *Wnt1* pathway regulators). Once the migratory cardiac neural crest subpopulation finally reaches the heart, they have traditionally been thought to participate in septation of the common outflow tract into separate aortic and pulmonary arteries. However, several studies have suggested these colonizing neural crest cells may also play additional unexpected roles during cardiovascular development and may even contribute to a crest-derived stem cell population. Studies in both mice and chick suggest they can also enter the heart from the venous inflow as well as the usual arterial outflow region, and may contribute to the adult semilunar and atrioventricular valves as well as part of the cardiac conduction system. Furthermore, although they are not usually thought to give rise to the cardiomyocyte lineage, neural crest cells in the zebrafish (*Danio rerio*) can contribute to the myocardium and may have different functions in a species-dependent context. Intriguingly, both ablation of chick and *Xenopus* premigratory neural crest cells, and a transgenic deletion of mouse neural crest cell migration or disruption of the normal mammalian neural crest gene expression profiles, disrupts ventral myocardial function and/or cardiomyocyte proliferation. Combined, this suggests that either the cardiac neural crest secrete factor/s that regulate myocardial proliferation, can signal to the epicardium to subsequently secrete a growth factor/s, or may even contribute directly to the heart. Although there are species differences between mouse, chick, and Xenopus during cardiac neural crest cell morphogenesis, recent data suggest mouse and chick are more similar to each other than to the zebrafish neural crest cell lineage. Several groups have used the genetically defined *Pax3* (*splotch*) mutant mice model to address the role of the cardiac neural crest lineage. Here we review the current literature, the neural crest-related role of the *Pax3* transcription factor, and discuss potential function/s of cardiac neural crest-derived cells during cardiovascular developmental remodeling.

## INTRODUCTION

Neural crest cells (NC) are a multipotent and transient migratory embryonic lineage that ultimately gives rise to an enormous array of different cell types, tissues, and organs[1]. The NC are required at different developmental stages for normal development of diverse organ systems, such as the peripheral and enteric nervous systems, facial skeleton, melanocytes, and cardiac outflow tract (OFT) septum. NC induction occurs in the neural folds at the dorsal aspect of the developing spinal cord (initially referred to as the neural tube). In response to interaction between the surface ectoderm and neural plate (which subsequently forms the neural tube), NC undergo epithelial-mesenchymal transformation (EMT), and then migrate (over and through non-NC lineages) and ultimately undergo differentiation along various specific developmental pathways at their sites of colonization[2]. Multiple local signals are thought to regulate the fate and function of these cells as they migrate to their terminal locations[3] (see Table 1). Different regions (loosely based on rostral-caudal neural tube variations) express different molecular expression profiles and can give rise to diverse NC-derived cell types. Due to the wide range of migration and the multistep process of NC morphogenesis in the embryo, they are especially vulnerable to both environmental and genetic disorders. Many congenital birth defects are thought to be due to aberrant NC morphogenesis[4].

## CNC SPECIFICATION, EMT, AND MIGRATION TOWARDS THE HEART

### Specification

Cardiac NC (CNC), which are a subpopulation of the NC, originate from the lower hindbrain between the otic placode and fourth somite[4,5,6], and undergo EMT and migrate towards the heart via the third, fourth, and sixth pharyngeal arches[7]. They are called CNC because this region of the neural tube provides mesenchymal cells to the heart and the great arteries. The NC-derived mesenchymal cells are often referred to as ectomesenchyme to discriminate them from “normal” mesenchymal cells that are derived from existing mesoderm, suggesting they are intrinsically different[8,9,10,11,12].

Although temporally defined via quail-chick chimeric analysis[13,14,15], chick microsurgical ablation of premigratory neural folds[4,16], and mouse Cre/loxP transgenic lineage mapping data in embryos that have their NC lineages permanently marked with a ß-galactosidase reporter[17,18,19] (see Fig. 1), identification of a CNC-specific inducing factor within the early neural tube has remained elusive. Surprisingly, despite the exquisite anterior-posterior patterning via overlapping homeobox gene expression in the neural tube, relatively few cardiovascular abnormalities have resulted from altered *Hox* gene expression[20]. To date, there is no CNC-restricted Cre lineage marker mouse line, thus all published lineage mapping data are the result of the simultaneous permanent marking of cranial, cardiac, and trunk NC progenitors. Most commonly used are the *P0-Cre, Wnt1-Cre,* and *Pax3-Cre* transgenic lines[17,18,19]. *Wnt1-Cre* is the earliest NC-restricted Cre line and provides both a useful lineage marker system (Fig. 1) and a means with which to target NC-related genes conditionally.

Although not restricted to the CNC-containing region of the neural tube, mutation of the Pax3 (*splotch*) transcription factor results in various CNC-related aortic arch and OFT defects[4,19,21]. As this model is 100% penetrant, it provides a useful genetically defined mouse model in which to study CNC cell morphogenesis. There are five *splotch* alleles with different mutations of the *Pax3* transcription factor: two of these alleles (*sp* and *sp*$^{2H}$) have provided a robust and morphologically well-characterized

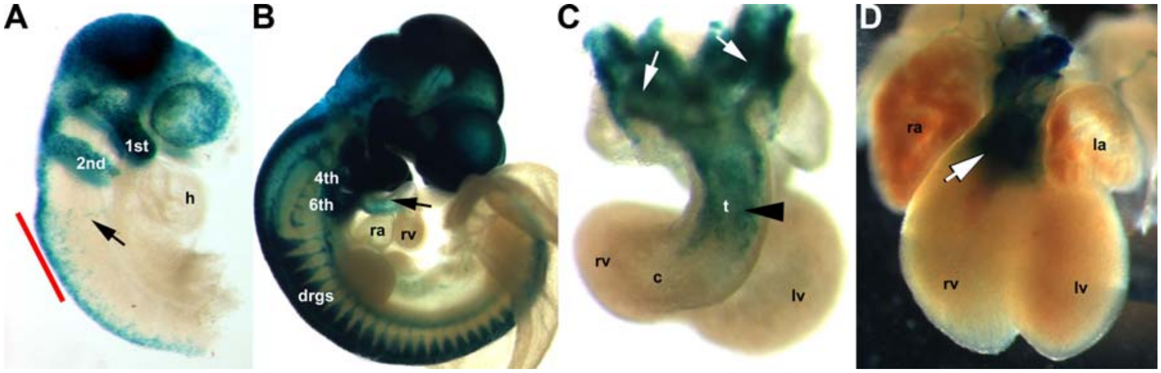

**FIGURE 1.** Specificity and efficiency of NC marking using the *Wnt1-Cre*[11] and *R26R* reporter Cre/loxP system. (A) Whole-mount staining of an E8.0 embryo. Labeling is seen in the dorsal neural tube, first and second pharyngeal arches (numbered), and migratory CNC (arrow). Location of CNC progenitors is indicated by red line. The OFT of the heart (h) is unlabeled, but is in obvious proximity to the pharyngeal arches. (B) Staining in an E11.0 embryo. Extensive labeling is seen in the head, dorsal neural tube, NC-derived dorsal root ganglia (drgs), all the pharyngeal arches (fourth and sixth numbered), and in CNC colonizing the OFT of the heart (arrow). (C) Isolated E11.0 heart. *LcZ* labeling can be seen in the aortic arch arteries (white arrows) and the truncus (t) of the OFT cushions (large arrowhead), but not in the conus (c). (D) E14 mature septated heart. Note robust *lacZ* reporter staining is present in the condensed mesenchyme of the OFT conus (arrow) and the anterior divided truncus, but is absent from the ventricles and atria. Abbreviations: rv, right ventricle; lv, left ventricle; ra, right atria; la, left atria.

model of aortic arch and OFT defects[21,22,23,24]. The $sp^{2H}$ homozygotes[25,26] die *in utero* and exhibit conotruncal septation defects: persistent truncus arteriosus (PTA) with obligatory perimembranous interventricular septal defect (VSD). The pathogenesis of the defect is due to the failure of the left sixth arch artery to persist, which usually gives rise to the pulmonary trunk (see Fig. 2). In addition, $sp^{2H}$ embryos exhibit defects within neural tube closure (spina bifida and excencephaly), melanocytes, and lack of limb musculature[27,28]. Significantly, *Pax3* mRNA is expressed within the neural tube, migratory NC cells and their derivatives (such as the thymus, thyroid, and dorsal root ganglia), somites, and melanocytes[29]; all structures are abnormal in $sp^{2H}$ homozygotes. In humans, haploin-sufficient *PAX3* mutations lead to Waardenburg syndrome, an autosomal-dominant disorder that consists of defects in NC-derived tissues and is characterized by pigmentation, hearing, and facioskeletal anomalies[30]. Cardiac defects have also been reported in some Waardenburg children[31,32].

Using the $sp^{2H}$ allele (*Pax3* homeodomain deleted), we have shown that expression of the NC marker *Ap2*$\alpha$ revealed extensive reduction in $sp^{2H}$ migratory CNC lineage. However, the rates of cell proliferation and apoptosis were unaffected and thus do not account for the observed $sp^{2H}$ CNC-associated heart defects[21]. Significantly, expression analysis revealed *Wnt1*, but not *Wnt3a*, is expressed at decreased levels within $sp^{2H}$ and the CNC fail to undergo normal NC progenitor proliferative expansion prior to migration while still in the neural folds. These data suggest the $sp^{2H}$ defect is intrinsic to the NC progenitors themselves and there is a decrease in the number of premigratory CNC that form. It appears this decrease in NC numbers is the primary defect that ultimately leads to a lack of a CNC-derived $sp^{2H}$ OFT septum. Both the *Wnt1* and *Wnt3a* cystein-rich secreted signaling molecules are expressed by NC precursors around the time of neural fold closure[33] and are thought to be essential for initial expansion of NC in the neural tube[34,35]. Double homozygous null *Wnt1/Wnt3a* mutant embryos have a significant reduction within NC-derived structures that do not seem to result from abnormalities within NC

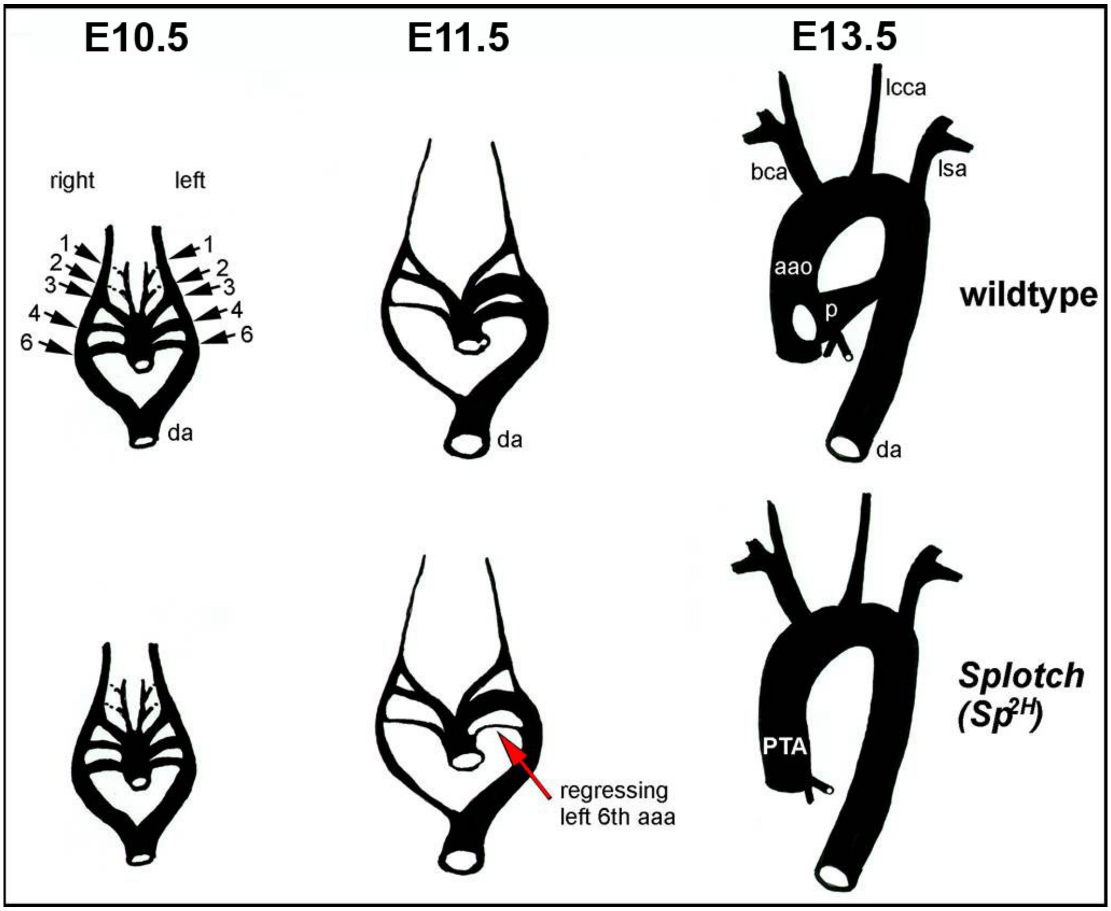

**FIGURE 2.** Schematic representation of the sequential changes that occur during remodeling of aortic (pharyngeal) arch arteries. Following ink injection analysis[58] of both age-matched wild-type and $sp^{2H}$ mutant littermate embryos, representative vascular casts were schematized. Upper panel indicates the normal pattern of regression and lower panel indicate the anomalous remodeling occurring within the $sp^{2H}$ mutant mouse. Normal remodeling. Note that there are initially six symmetrical arteries attached to the paired dorsal aorta (da) that are remodeled to give rise to a separate ascending aorta (aao; partly derived from the left fourth artery) and pulmonary trunk (p; derived from the left sixth artery) with two pulmonary arteries attached. Elements of the brachiocephalic artery (bca) are derived from the right fourth/sixth arteries, the left common carotid artery (lcca) is derived from the left third artery, and the left subclavian (lsa) is derived from the seventh intersegmental artery. *$Sp^{2H}$* remodeling. Note there are initially six symmetrical arteries, but that the left sixth (which normally gives rise to the pulmonary trunk) abnormally regresses, resulting in only a single OFT vessel and PTA.

emigration, migration, survival, or differentiation, but rather a lack of NC expansion in the neural tube[34]. Significantly, the *sp* cardiac phenotype can be rescued by transgenic overexpression of *Pax3* under the control of a 1.6-kb neural tube and early NC-specific *Pax3* promoter that is not expressed within somites[36]. Transgenically rescued *sp* homozygotes survive until birth, at which time they succumb to respiratory failure secondary to absence of a muscular diaphragm. *Pax3*-deficient somites are capable of supporting proper CNC migration and function, indicating a cell autonomous role for *Pax3* during NC morphogenesis. The critical role of *Pax3* in NC progenitor expansion is supported by data that *sp* homozygote heart defects can also be genetically "rescued" by crossing the *sp* mutants with the viable *Msx2* homeobox-containing null mice[37]. Double *Pax3-Msx2* survive until birth and then die due to respiratory failure. These transgenic rescue experiments elegantly demonstrate that *Pax3* is required for the repression of *Msx2* expression within the dorsal neural tube, and that appropriate specification of CNC progenitors is a critical first step. Given that *Msx2* is a well-documented regulator of Bmp signaling, it will be interesting to determine what role the TGFβ superfamily plays during CNC specification.

## EMT

Although little is known about CNC specification, even less is known about CNC-specific EMT, although the zinc finger genes, *Slug, Snail, Id2*, and *Pinch-1,* seem to be involved in specifying EMT competence[5,38,39,40,41]. The details, especially the temporal order of events in NC EMT, vary between different species and between different axial levels, but several important features have emerged[38]. EMT is strongly associated with a decrease in cell-cell adhesion, particularly with loss of N-cadherin on the surface of NC cells at the time of onset of emigration[42]. Similarly, N-Cam adhesion molecule also declines on NC cells. The surrounding extracellular matrix is also important, as EMT-related changes have been found in several matrix receptors (i.e., integrins), while the nature of the matrix itself is also modified. Changes in Rho family GTP-binding proteins (*RhoB*), cell shape, and in cell motility also occur at the time of EMT, consistent with changes in the cytoskeleton[43], and these concerted changes can be triggered by TGFβ superfamily growth factors. Given the exciting identification of *Sox9* transcription factor as a master regulator of trunk NC induction, survival, and delamination, and the role of the winged-helix transcription factor *FoxD3* as a regulator of cell-cell adhesion molecules required for subsequent trunk NC migration[44], it will be interesting to see if a similar mechanism can be identified for the CNC lineage.

## Migration

CNC migration and homing to the third, fourth, and sixth pharyngeal arches and cardiac OFT niches occurs during a well-defined developmental time window and along characteristic circumpharyngeal migration pathways[15,16,19,45]. Adhesion molecules, such as integrins, are involved in the interaction of CNC with the extracellular matrix, while cadherins and gap junctions allow CNC to interact with each other during their migration. Migration of CNC to the heart has been shown to be modulated by interactions mediated by connexin43 (*Cx43α1*) gap junction membrane channels between cells. CNC express Cx43α1, are functionally-coupled[46], and *Cx43α1* knockout mice die at birth with conotruncal heart malformations, outflow obstructions, and coronary anomalies[47,48]. CNC motility is dependent on the level of Cx43α1 function, as loss of Cx43α1 inhibits NC cell migration, while overexpression enhances migration. Recent chick and mouse studies suggest that Cx43α1 may modulate cell motility, via mediating cross-talk with cell signaling pathways (vinculin and other actin-binding proteins) that regulate polarized cell movement essential for the directional migration of CNC[49]. In parallel, gap junction formation has been shown to be dependent on cadherin-mediated cell–cell adhesion[50] and a NC-restricted knockout of *N-cadherin* has recently been shown to result in CNC-related mouse embryonic OFT defects[51]. Significantly, using *N-cadherin* null mouse NC cells, it has been demonstrated that *N-cadherin* can modulate NC cell motility by engaging in dynamic cross-talk with the cells' locomotory apparatus via p120 catenin signaling[50]. In *Xenopus*, the canonical (β-catenin dependant) *Wnt* signaling pathway is thought to be important for NC cell induction, while the noncanonical (planer cell polarity) signaling is thought to regulate NC migration[52]. Using time-lapse analysis on cultured NC cells (either control or cells injected with the dominant negative form of *Wnt11* mRNA), it was shown that the noncanonical pathway controls migration by directing the stabilization of cell protrusions necessary for locomotion. When the *Wnt* noncanonical pathway is disrupted, more CNC cells have filopadia protrusions, but they are significantly less polarized. Thus, *Wnt11* is thought to be the activating ligand of the *Wnt* noncanonical pathway and is expressed adjacent to the CNC prior to migration. This link is further strengthened as it was also shown that the *Wnt11* receptor (*Frizzled*) is expressed in a subpopulation of both premigratory and migratory *Xenopus* NC cells[52].

## AORTIC ARCH ARTERY REMODELING DEFECTS ASSOCIATED WITH LACK OF NC

### Aortic Arch Artery Remodeling

During normal cardiovascular development, the early OFT is a single vessel that branches at the aortic sac into the bilaterally symmetric third, fourth, and sixth aortic arch arteries present within the pharyngeal arches. Remodeling of these transient symmetrical arch arteries into the definitive adult left-sided aortic arch vascular pattern involves the asymmetrically programmed regression and persistence of specific arch arteries (see Fig. 2). The third arch arteries give rise to common carotid arteries, fourth arch arteries contribute to the formation of the distal part of the aortic arch, the brachiocephalic artery and a proximal part of the right subclavian artery, while the sixth arch arteries contribute to the ductus arteriosus and the proximal parts of the pulmonary arteries. The final arrangement and morphology of these great vessels requires reciprocal signaling between the endothelial cells lining the pharyngeal arch arteries[53], the surrounding NC-derived smooth muscle and mesenchyme[54,55], and the endoderm[56,57]. Anomalous remodeling underlies a wide variety of congenital heart defects including: PTA, coarctation and interruption of the aortic arch, double aortic arch, right aortic arch, and abnormal origin of the right subclavian (pathogenesis of these defects reviewed in Conway et al.[58]). PTA arises when the arterial trunk fails to be divided to form a separate pulmonary artery and aorta. Despite the presence of many different mouse mutant models of anomalous aortic arch artery development (several within the DiGeorge region), the precise role of the CNC during aortic arch artery morphogenesis is not well understood[7,22,59].

### Colonization of Pharyngeal Arches

The pharyngeal arches are initially composed of mesenchyme (mesodermally derived), which is surrounded externally by ectoderm and internally by endoderm. Subsequent to CNC colonization, most of the mesenchyme is CNC derived, apart from the original mesodermal core that lies adjacent to the aortic arch arteries[18]. In addition to a variety of complex gene expression profiles[6], the mesenchyme of both the anterior and posterior pharyngeal arches contain different partially restricted intermediate cell types derived from the NC[60], suggesting that the pharyngeal arches are segmentally patterned. CNC-derived mesenchyme subsequently condense and differentiate into fibrous connective tissue that contributes to vascular stabilization of the great arteries[7], while other NC populate the cardiac ganglia[15]. A substantial population of CNC-derived cells in the pharyngeal arches remains associated with the pharyngeal arch arteries, and constitutes the smooth muscle layer that surrounds these vessels as they become reorganized into the arch of the aorta, the ductus arteriosus, and the proximal segments of the carotid arteries[18,61]. Comparison of chick and mouse CNC migration patterns using a combined *Cx43-lacZ* mouse transgenic marker and quail-chick chimeras clearly demonstrated that CNC migration is similar within the pharyngeal region[15]. The similarities include the formation of a sheath around the aortic arch arteries and population of the cardiac ganglia. However, it is still unclear what role the CNC play during asymmetric remodeling and whether they play an instructive role or secondarily respond to other stimuli (for instance differential blood flow patterns and/or neural innervation). Migration of a subpopulation of CNC continues on into the cardiac OFT where they populate the conotruncal cushions[4,23,24] and, by currently unknown mechanisms, they participate in OFT septation[62]. NC from this same region of the mouse neural tube also give rise to cells of the thymus, thyroid, and parathyroid glands[18,19]. There is extensive experimental evidence (little of which is genetic) suggesting that the various steps in NC morphogenesis and cell fate are influenced by cell-cell and cell-matrix adhesions[63,64] and the environment through which they migrate[3]. Consequently, the pharynx is a likely source of important instructive signals for the migrating CNC. In fact, the primary fate of the CNC lineage is to differentiate into the smooth muscle of the aortic arch arteries and cardiac OFT[1,18,61], and require NC autonomous expression of *Pdgf receptor α*[65], *Alk2* receptor[66], and *Alk5* receptor expression[67].

Although our understanding of the role of the CNC remains mostly descriptive, some insights into the cellular processes have come from experimental manipulation of chick embryos[4,11] and the genetic processes from mouse, zebrafish, and *Xenopus* mutants[68]. In chick embryos, neural fold/NC ablation[4], *Hox* antisense experiments[20], teratogenic retinoic acid exposure[69], and hemodynamic perturbations[70] have all caused fourth and sixth pharyngeal arch abnormalities. Similarly in mouse, teratogenic exposure to haloacetic acids[71], ethanol[72], and retinoic acid gives rise to arch abnormalities. Related or analogous pathological defects of the mouse fourth and sixth pharyngeal arches and OFT are also seen when a large number of genes have been transgenically altered[73,74]. For instance, the $sp^{2H}$ homozygotes[23,24,25,26] die *in utero* and exhibit PTA with obligatory perimembranous VSD due to the failure of the left sixth arch artery (which would normally form the main pulmonary artery segment) to persist. Using ink injection casts to assess third, fourth, and sixth pharyngeal arch artery remodeling, we found that all three pairs of arch arteries were formed, but that both the left and right sixth arch arteries underwent vascularization and disappear in $sp^{2H}$ homozygotes when compared to wild-type littermates (see Fig. 2). This suggests that pulmonary atresia underlies the OFT defects and that only one vessel remains that exits the heart. We have also shown that presumptive $sp^{2H}$ homozygote CNC stem cells fail to undergo normal progenitor expansion within the neural tube and consequently insufficient CNC colonize the pharyngeal arches and OFT[21]. This suggests that normal aortic arch artery remodeling is partly dependent on a particular threshold number requirement of colonizing NC. Similarly, when any of the aforementioned manipulations result in elevated CNC apoptosis and/or suppressed NC proliferation, anomalous remodeling of the aortic arch arteries and/or vascular regression is often a downstream consequence[73,74].

## Coordinated CNC-Derived Mesenchyme Differentiation is Required for Remodeling of the Arterial Tree

Mutational analysis has identified a large number of genes required for morphogenetic and inductive processes involving the mouse CNC[4,22,68]. These genes appear to involve several different pathways acting in parallel or in series with one another, but there is currently no simple pathway that unifies all the available data. Some genes, including *Pax3* and *Ap2α*, are expressed in the migrating CNC as they relocate to their positions around the great vessels and OFT[21,23]. More recent evidence suggests that these interactions, and the programming of the CNC, are mediated by transcription factors including *Foxc1/Foxc2*, *Hand1/Hand2,* and *Tbx1* and other genes on human chromosome 22q11[68,75,76,77]. Another set of genes is expressed within the developing vasculature itself, and may play a role in vessel formation, stabilization, and remodeling (*Vegf-A*[78]; *neuropilin-1*[79], or within the interacting mesenchymal cells (*Mef2c*[80]; *Tissue factor*[81]). This has led to the idea that the control over vascular assembly resides within the connective tissue–forming NC-derived mesenchyme. In support of these reciprocal interactions, Noden[82] found similar results using quail-chick transplantation. Finally, there is still another set of genes that is expressed in the pharyngeal arches themselves, and may play a role in mediating interactions between the arch epithelia (ectoderm and endoderm), mesenchyme, and endothelial vessels (*Endothelin-1*[83]; *Ece-1*[53]; *EtA*[84]; *Semaphorin3c*[85]). Interestingly, *TBX1*, a gene associated with DiGeorge syndrome, is not expressed in the CNC, but in the adjacent mesendoderm of the pharyngeal arches[86]. Furthermore, expression of the secreted growth factor *Fgf8* is diminished in *Tbx1*-expressing cells from *Tbx1* mutant mice[57], and mice deficient in *Fgf8* exhibit elevated levels of CNC cell apoptosis and the typical DiGeorge syndrome interrupted aortic arch phenotype[87,88]. However, it is not currently known whether CNC cells are direct targets of Fgf8 or whether its effect is indirect. Although down-regulation of Bmp signaling in pharyngeal endoderm seems to be a prerequisite for CNC cell survival[86,89,90], NC-restricted deletion of *Bmp type I receptor Alk2*[61] results in abnormal maturation of the aortic arch arteries and PTA. Thus, it is possible that Fgf and TGFβ

superfamily signaling pathways converge to control CNC cell fate during both aortic arch artery remodeling and cardiac OFT morphogenesis[67].

Cell fate studies using a transgenic *Cre/loxP* cell marking technique have demonstrated that the mere presence or absence of CNC is not sufficient to cause remodeling, as there do not appear to be any differences in the distribution between those arteries that persist vs. those that regress[18]. This suggests that different left-right signals required for remodeling must be carried by the colonizing CNC or are present within the mesodermal core, endothelial cells lining the arch arteries, or within the pharyngeal pouch/cleft endoderm. The origin and identity of these signal/s is currently unknown, but may involve retinoic acid levels[91] or differential activity of ion channels (*Polycystin-2*[92]) giving rise to unidirectional transfer through gap junctions, resulting in asymmetric gene expression[93]. Currently, *Pitx2[*94] is the only known gene asymmetrically expressed within the pharyngeal arch mesoderm, and it has been shown that an isoform-specific deletion of *Pitx2c* results in abnormal patterning of the aortic arch vessels[94]. Thus, it is well established that CNC can provide structural integrity and may also carry instructive signals required for aortic arch remodeling. Disruption of this signaling leads to defects in the interactions between postmigratory CNC and the endothelium of the great vessels and OFT. Collectively, these results support a model in which epithelia/endothelia of the arches signals to NC-derived mesenchyme (possibly via processed *Et-1*, *Semaphorin,* and/or *Vegf*) possibly through gap junctions. However, it is unclear exactly what the function of the NC is within the arches and OFT, how they differentiate into connective tissue, and which genes respond to the various CNC-mediated differentiation signals.

## ROLE OF CNC IN OFT DEVELOPMENT

### OFT Elongation and Septation

A subpopulation of the CNC continues migration and colonizes the common OFT endocardial cushions. Although the CNC cell patterns in the pharyngeal region are similar in mouse and chick embryos, a couple of notable differences have been reported as they enter the OFT cushions prior to septation[15]. In quail-chick chimeras, the CNC enter by two distinct routes, subendocardially and submyocardially. This contrasts with the single subendocardial entry route of *Cx43-lacZ* marked mouse CNC. Furthermore, migration of CNC cells into the mouse OFT extends all the way to the distal conus, whereas they only extended into the conus in the chick and do not reach the conotruncal transition. These CNC patterning differences between species may be due to innate morphological differences between mammals and birds, as well as differences in timing of some of the developmental events in cardiovascular development[15]. Elongation of the OFT is a prerequisite for correct looping, complete rotation, and appropriate alignment during OFT septation. Both the CNC and anterior heart field (AHF) lineages are required for OFT elongation, as the CNC contribute to the existing conal endocardially derived OFT mesenchymal cushions, while the AHF cells contribute to the OFT myocardial cuff and most of the right ventricle[95]. Following CNC cell colonization of the truncal cushions, the common OFT is divided into a separate pulmonary artery and the aorta. This is accomplished by the development of the endocardial cushion conotruncal ridges, which grow caudally in a spiral fashion, resulting in posteriolateral realignment of a separate aorta and anteriomedial realignment of a separate pulmonary artery. This spiral septum fuses with the bulbar ridges, which, together with proliferation of the inferior endocardial cushion, close the interventricular septum (failure to do so results in VSD). Chick studies have shown that labeled CNC cells can undergo apoptosis on completion of septation[11,96], however, there are limited data as to whether this also occurs (and to what extent) during mammalian OFT septation. Martinsen et al. discussed the observation that there is an extension of the CNC to the rim of the right ventricular outflow tract[98] and that the ultimate fate of these deep CNC cells may be apoptosis[97,98,99]. This would explain why there are no NC-derived septal structures below semilunar valve level[97,99]. Furthermore, it is hypothesized that as the NC under go apoptosis, they may release or mobilize growth factors[97]. The population of deep migrating cardiac NC, whose fate is cell death, may be marked by *Id2,* which also marks the secondary heart field and ganglia of the anterior parasympathetic plexus[98]. Similarly, mouse CNC cells in the OFT colocalize in a knot of TUNEL-positive condensed mesenchyme that transiently express a Wnt receptor *Frizzled-2*[100], suggesting a role in remodeling and patterning during septation. However, using transgenic mouse *Cre/loxP* cell marking techniques, it has been shown that CNC cells populate the conotruncal cushions and contribute to cardiac tissue found at later stages of fetal heart development and to a lesser extent in the adult heart[17,18,19]. Thus, not all the mouse CNC cells undergo apoptosis and a distinct population can be found in the mature heart. Initially, *Wnt1-Cre* permanently labeled mesenchymal cells project from the aortic sac in an unbroken stream through the conotruncal region, up to the junction of the conus with the wall of the right ventricle[18]. However, during septation and fusion of the spiral septum, the widely distributed CNC become localized within a thin subendothelial layer along the seam of fusion. Thus, it appears that the mass of CNC-derived cells that constitutes the early aorticopulmonary and conotruncal mesenchyme mostly dies or is overgrown as septal formation is completed[18].

## Remodeling of the OFT Septum

Once in the OFT cushions, it has been suggested the CNC cells are involved in fusion and subsequent myocardialization of the proximal OFT, giving rise to the muscular outlet septum of the heart[101]. Rather than transformation of endocardial cushion cells (either endothelial or NC derived), it is thought that normal myocardialization of the cushions is caused by a redistribution of existing cardiomyocytes and regulated by (as yet unknown) secreted factors[102]. Myocardialization is only seen to occur in cushion mesenchyme, which is found inside the heart tube, and never in the epicardial mesenchyme, which covers the heart at the outside. Thus, the observation that disruption of one of the noncanonical *Wnt* pathway core planer polarity molecules leads to OFT misalignment defects is exciting[103,104], as this suggests cytoskeletal changes that affect cell adhesion, motility, and polarity do play a role during OFT septation. Similarly, NC-restricted N-cadherin deletion results in OFT remodeling defects and PTA[105]. The N-cadherin null OFT cushions exhibit misshapen (more rounded) CNC cells with fewer cell-cell contacts when compared with wild-type littermates, and undergo elevated levels of apoptosis, indicating that N-cadherin is required for CNC cell survival in the OFT. However, the precise role of the CNC cells and the molecular mechanisms responsible for the normal remodeling of the initial common OFT into two asymmetrical OFT vessels is largely unknown. For instance, it is unclear exactly how CNC differentiate into connective tissue and what CNC-mediated differentiation signals are expressed during septation. Finally, although there are several markers expressed during CNC cell migration into the arches and up to the OFT[21,23], there are currently no markers/candidates that are expressed in the CNC cells as they enter, undergo differentiation, and/or reside within it. This makes the elucidation of their role and function once they reach the heart elusive. This is a particular drawback when considering the ultimate role of the OFT mesenchymal cushion cells in OFT morphogenesis and pathogenesis of congenital heart defects. Thus, it is critical to find new genetic targets that either continue to be expressed as the CNC cells colonize the heart or get turned on by the colonizing CNC cells in the OFT. The field needs a broader array of molecular markers to help dissect both the earlier and later steps of CNC cell colonization of OFT[6], as well as a better understanding of the signals seen by the CNC cells that are involved in aortic arch artery remodeling and formation of OFT septum.

## Unexpected Roles of NC

Given that the vast majority of CNC molecular markers, both mRNA and protein, are switched off as CNC colonize the OFT cushions and our reliance is on only a couple of transgenic marking techniques, the ultimate fate of the CNC remains uncertain. While most studies have supported the role of CNC in

OFT septation, several mouse and chick lineage marking experiments have also suggested a wider unexpected role for the CNC deep within the heart. Using retroviral labeling of the neural tube prior to EMT, it has been shown that CNC cells can enter the embryonic chick heart from two areas. First is the well-known pathway through the pharyngeal arches and into the arterial pole. The second entry is via the venous pole and dorsal mesocardium[106]. The arrival of NC to the venous pole occurs later in development than those in the OFT, and they can migrate to the atrioventricular cushions and surround the conduction system. Once venous pole cells reach these destinations, they appear to undergo apoptosis coincident with physiological function changes in the conduction system. Complimentary *Cre/loxP* mouse lineage mapping studies using *Wnt1-Cre* have similarly reported that NC cells can enter the heart from both venous and arterial poles[107]. The colonizing arterial pole CNC are associated with the cardiac conduction system and can contribute to bundle branches, while the venous pole cells contribute to the sinoatrial and atrioventricular nodes[107]. Using the same *Wnt1-Cre* (in parallel with complimentary *P0-Cre* mice), in conjunction with both the β-galactosidase and EGFP *Cre/loxP* lineage mapping system, a recent report demonstrated that a significant number of NC was seen to contribute to the adult semilunar and atrioventricular valves[108]. These studies also showed that NC enter from both the venous and arterial poles and can contribute to the proximal conduction system at late developmental stages. Furthermore, the NC in the atrioventricular valves simultaneously expressed several NC-related differentiation markers, suggesting some NC in the embryonic heart are not fully differentiated and may remain multipotent[108]. Two possible reasons for these apparent discrepancies with previous reports[11] and our own lineage mapping data (Fig. 1) are thought to be mixed vs. inbred genetic background effects that might allow CNC to migrate further than has previously been seen by others, and/or differences in Cre expression levels and types of indicator mice used (i.e., ROSA26R vs. CAGCAT-EGFP mice). Using the NC-restricted *P0-Cre* lineage marker mice crossed to floxed EGFP indicator line, one group has intriguingly shown that a small population of EGFP positive NC in the heart can colocalize with known stem cell markers[109]. Retroviral labeling of the chick neural tube was also used by Sohal et al.[110], who found that neuroepithelial cells may emigrate from the ventral side of the neural tube (termed VENT cells) and can give rise to numerous cell types in the developing cardiovascular system. Based on cell-labeling studies in the hindbrain of avian embryos using replication-deficient retroviral vectors containing *LacZ* to permanently label their progeny, VENT cell emigration is thought to occur after CNC emigration has ceased[110]. However, the colonization of the heart was inferred retrospectively after examining numerous embryos harvested at different stages. Given these shortcomings and the lack of any specific molecular marker, it is unclear whether there is a contribution of ventral neural tube cells to the heart[111,112].

Surprisingly, identification of these various unexpected roles of NC have all used many of the same techniques and reagents that were initially employed to define the traditional role of the CNC lineage during OFT septation. Although each of these studies is in agreement as to the fact that the majority of the CNC colonize the pharyngeal arches and OFT, the fact that they do all report a lesser (but detectable) contribution to the heart is significant. Our own studies with the *Wnt1-Cre x R26R* reporter system match those reported by Jiang et al.[18] and have thus far failed to detect venous pole entry or labeled CNC in the valves (unpublished). Intriguingly, Kirby and colleagues have shown that double ablation of the nodose placode and the CNC yields more consistently PTA[113]. Thus, nodose placode cells could be a population of cells that compensates for the CNC that are not always present in the unexpected lineages[113].This suggests that the degree to which these unexpected derivatives are detectable may depend on the various genetic backgrounds of the mice lines, as it is unlikely that variable Cre-mediated recombination efficiencies could account for these consistent differences. Although these data are difficult to reconcile with many of the traditional CNC-related phenotypes seen with various experimental and/or genetic NC targeting studies, the known pluripotent nature of NC means that unexpected derivatives warrant further investigation. Collectively, these intriguing results suggest that, depending on the setting of experimental and/or genetic CNC abnormalities present, and the genetic background used, NC can make their way into the rest of the heart, but they do not necessarily do so. Given that these derivatives do not always contribute to these unexpected lineages and are not always present, it remains to be seen what role they play when present.

## CNC CELL EFFECTS ON MYOCARDIAL DEVELOPMENT

### *Pax3* Mouse Model

In embryos homozygous for two different *splotch* alleles (*sp* and $sp^{2H}$) that each contain *Pax3* mutations, all the mutants exhibit PTA (with obligatory VSDs) and die mid-gestation. The presence alone of PTA/VSD should not cause *in utero* lethality[58], as the systemic and pulmonary circulations are not required to be separate until birth, thus additional causes of lethality have been investigated. Significantly, 100% of the $sp^{2H}$ homozygous mutant embryos die ~E14.0 and exhibit edema, pooling of blood in caval veins, and engorgement of the fetal liver, which are all suggestive of poor cardiac function[23,24]. Deficiencies in myocardial $Ca^{2+}$ handling further compromise cardiac function, as there is abnormal excitation-contraction coupling in the $sp^{2H}$ mutant cardiomyocytes[24]. It has been proposed that this myocardial defect is an indirect consequence of the reduced numbers of migrating CNC[37] because they are not generally thought to contribute to the myocardium[18]. The *sp* homozygous embryos also die ~E14.0 due to cardiac failure, but the *sp* hearts also present with a thinned myocardium and an absent compact zone[36] (see Fig. 3). Using subtractive hybridization to identify mRNA transcripts whose expression is enhanced between E10.5–13.5 in normal hearts, it has been shown that *p57Kip2* (which encodes a cyclin-dependent kinase inhibitor of the *p21* family) is up-regulated and ectopically expressed in the myocardium of *sp* embryos[114]. As the *sp* cardiac OFT and thinned myocardial phenotype can be rescued by transgenic overexpression of neural tube/NC-restricted *Pax3*[36], this suggests that the *sp* myocardial defects are CNC-related. Similarly, *Pax3-FKHR* knockin heterozygous mice are not viable and 100% die ~birth due to presence of VSD and exhibit cardiac insufficiency, and a grossly enlarged septum with a dilated OFT[115]. It is currently unclear if the *Pax3-FKHR* knockin is acting as a "dominant-negative" mutation affecting the heart (as *Pax3* itself has not been thought to play a role in myocardial morphogenesis) or if the *Pax3* mutation directly affects the heart (as Pax3 protein is detectable within isolated valvular cells in the E16.5 heart). Although not identical, it is interesting to note that both the *Pax3-FKHR* and $sp^{2H}$ mutants lack the C-terminal portion of the homeodomain that modulates DNA binding activity and controls specificity of target sequences[116]. As $sp^{2H}$ hearts have an intact compact layer, but still die *in utero*, we assessed trabecular and compact layer-restricted gene expression (Fig. 4). In contrast to *sp* mutants[114], $sp^{2H}$ mutants express normal levels and patterns of *Anf, Bmp10, p57Kip2* and *N-myc*, suggesting that lack of normal CNC colonization can affect different myocardial maturation signaling pathways that ultimately result in poor cardiac function and lethality.

Although a direct role for *Pax3* during cardiomyocyte morphogenesis remains elusive, Schafer et al.[117] have suggested that the *Lbx1* homeodomain-containing transcription factor and *Pax3* are involved in a regulatory feedback loop to repress each other indirectly within the ventricle. A small population of Lbx1 expressing NC migrate from the neural tube into the caudal pharyngeal arches and on into the OFT. *Lbx1* null embryos have cardiac looping defects, alterations in connexin gene expression, and hyperplasia of the myocardium[117]. *Lbx1* is up-regulated in $sp^{2H}$ mutant ventricles, suggesting that *Lbx1* null CNC are not programmed correctly and can disrupt heart development, resulting in cardiac malformations[117]. Complex embryological defects, including CNC and myocardial dysfunction, can be experimentally induced via prenatal exposure to the herbicide nitrofen[118]. Correlating with the aforementioned data, *Pax3* mRNA is decreased in rat embryo hearts that exhibit CNC-associated nitrofen-induced defects. As myocardial maturation is known to be regulated by adjacent epicardially derived signals[119,120], and as *Pax3* is not thought to be normally expressed by cardiomyocytes[121], these data suggest that a direct contribution by the CNC to the epicardium is an intriguing possibility. Although *Wnt1-Cre* expression has been associated exclusively with the NC and derivatives[18,122], the epicardium of

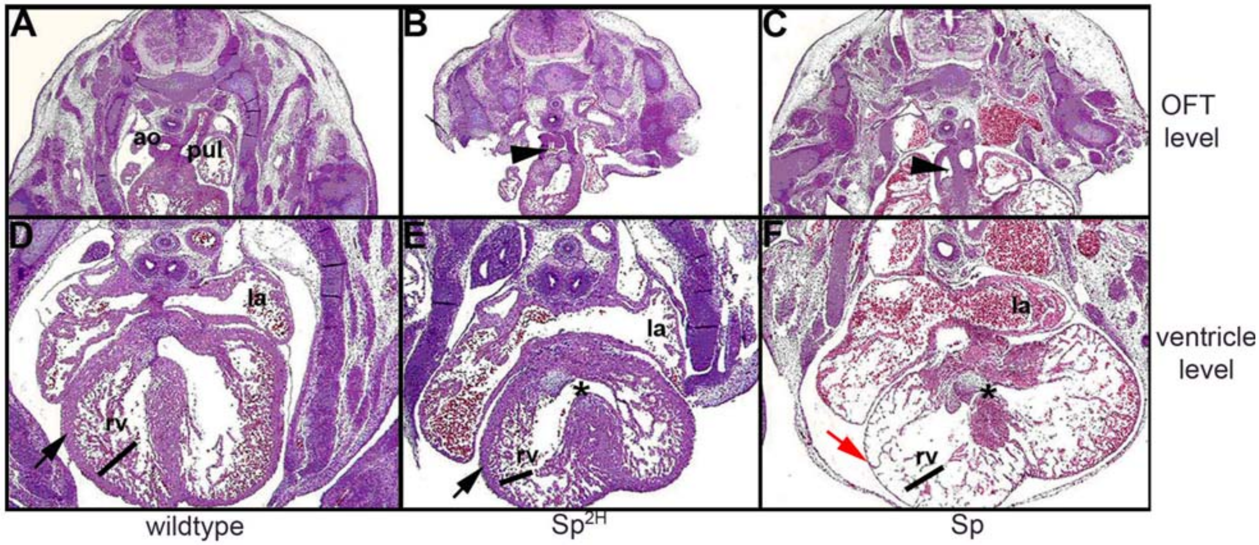

**FIGURE 3.** Histological analysis of the E13.5 ventricular myocardium in wild-type and pax3 allelic mouse mutants. (A and D) Wild-type, (B and E) *sp*$^{2H}$, and (C and F) *sp* embryonic hearts transversely sectioned. Note that both the *sp*$^{2H}$ and *sp* homozygous mutants have OFT defects (arrowheads) and VSDs (indicated by *), but that only the *sp* myocardium is abnormally thin. Trabeculation (indicated by bar in right ventricles; rv) is still present in *sp* mutants (although sparse), but the highly proliferative *sp* compact layer (indicated by red arrow) is severely diminished when compared to *sp*$^{2H}$ and wild-type littermates (arrows in D and E). Abbreviations: ao, aorta; pul, pulmonary artery.

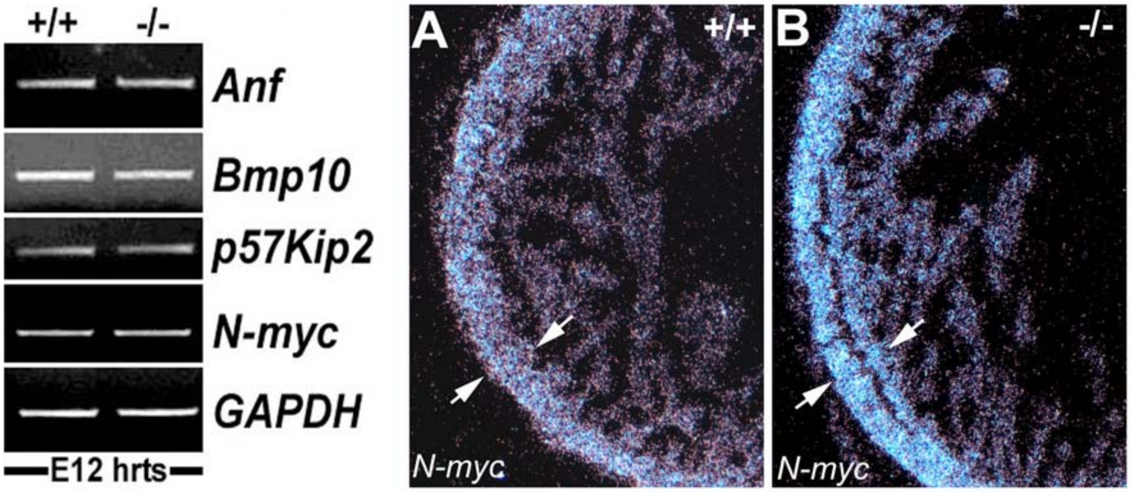

**FIGURE 4.** Molecular marker analysis of the E12 ventricular myocardium. (Left panel) RT-PCR analysis of trabeculae and compact layer-specific marker expression in wild-type and *sp*$^{2H}$ mouse mutant hearts. Expression levels of the trabecular marker genes *atrial natriuretic factor (Anf*), *bone morphogenetic factor 10 (Bmp10*), and *p57Kip2* are unaffected in homozygous *sp*$^{2H}$ hearts when normalized with *GAPDH* housekeeping gene expression. Similarly, expression of *N-myc*, a marker of compact myocardium, is normally expressed in mutant hearts. (Right panel) Radioactive *in situ* hybridization analysis of *N-myc* expression patterns supports the RT-PCR data and reveals that the *sp*$^{2H}$ compact layer (B) is present and appropriately expresses *N-myc* in the proliferative compact myocardial layer (indicated by arrows).

some *Wnt1-Cre x R26R* embryos has been reported to contain a small population of recombined cells[123] that could interact with the adjacent myocardium. In order to address these unanswered questions, we generated a “tissue-specific” targeted knockout of *Pax3*, that in conjunction with cardiomyocyte and epicardially restricted *Cre* mice, will enable us to begin testing whether the observed poor *sp*$^{2H}$ cardiac dysfunction is due to either null primary effects of the Pax3 mutation in the cardiomyocytes or secondarily due to earlier CNC and/or epicardial effects. These lineage-restricted knockout approaches will also enable us to determine whether abnormal CNC morphogenesis is sufficient to give rise to abnormal aortic arch morphogenesis, or whether myocardial dysfunction is additionally required.

## Direct or Indirect Effects on Cardiomyocyte Proliferation?

Similar to the *Pax3* mouse mutants studies, Kirby and colleagues have also shown that premigratory CNC ablation in chick consistently results in myocardial dysfunction prior to the arrival of the CNC within the heart, and have suggested that these early effects on the heart are due to a prolonged release of secreted growth factors (FGFs, etc.) by the pharyngeal endoderm, which are normally involved in the induction of cardiac mesoderm[15]. These FGF signals are proposed to suppress chick myocardial development and calcium transients/contraction, and alter myocardial proliferation/differentiation in the absence of CNC[15,124], but are prevented from disrupting cardiomyocyte morphogenesis when the full complement of CNC are present in the pharyngeal arches and OFT septum. These data suggest that the endocardium can indirectly have deleterious effects on myocardial maturation, as a consequence of absent CNC colonization.

Genetic evidence also indicates that normal CNC morphogenesis is necessary for normal myocardial development and in utero viability. Conditional deletion of Bmp receptor1a specifically in the NC using *Wnt1-Cre* results in defective myocardial formation, as well as a shorter OFT that fails to septate[123]. These mutant embryos die ~E12 with acute heart failure and exhibit a notable lack of ventricular myocardial proliferation. Lineage-tracing experiments suggested that a small population of permanently marked NC might be able to migrate to the epicardium[123]. Similarly, *Wnt1-Cre* conditional deletion of N-cadherin results in embryonic lethality ~E13 and OFT remodeling defects that resulted in PTA in the majority of the mutants[105]. N-cadherin NC-specific mutants are thought to die due to a thinned ventricular myocardium and detachment of myocardium from adjacent epicardium. Normally, the epicardium expands in an epithelial sheet to cover the ventricular surface, from where epicardial cells invade the underlying myocardium. Given that a few CNC can populate the epicardium[123], the reduction in ventricular wall thickness in both these NC-restricted mutants could be due to either a direct effect on the NC-derived epicardial cells that invade the myocardium (like other epicardial cells); or they could be secondarily due to loss of epicardial-myocardial signaling or Bmp receptor1a/N-cadherin insufficiency in the CNC and their subsequent inability to effectively colonize the OFT. It has been suggested that perhaps the CNC produce a factor that stimulates myocardial proliferation, and when inappropriately specified, they are unable to respond to OFT-restricted stimuli and consequently myocardial proliferation is reduced[123].

A second cell lineage, the AHF, has also been shown to play a major role during OFT development and septation. The AHF in mouse includes the early pharyngeal core arch mesoderm and splanchnic mesoderm, which overlie the ventral pharyngeal endoderm and can be identified prior to NC emigration within the primary heart field as early as the cardiac-crescent stage[125,126]. As AHF cells contribute to definitive OFT myocardium as well as to the right ventricle and some endocardium[127,128], inappropriate cardiomyocyte specification of the AHF lineage could subsequently result in compromised myocardial development. Given that the AHF and CNC may be interdependent, because surgical ablation of either CNC[10] or AHF[129] results in changes in OFT length, myocardial dysfunction could possibly result from inappropriate CNC-AHF cross-talk. Similarly, recent lineage-specific deletion of the Shh morphogen has elegantly shown that a Shh pharyngeal endodermal signal is directly required by both the AHF and CNC for normal OFT morphogenesis[130]. The possibility of AHF and CNC interactions suggest that communication and dynamic intracellular signaling among multiple lineages may be crucial during CNC colonization of the pharyngeal aortic arches and OFT development and septation.

## CNC Species-Specific Differences

In contrast to their mammalian and chick CNC counterparts, zebrafish CNC originate more rostrally along the neural tube, and can contribute to the myocardium and may have different functions in a species-dependent context[131]. Given the absence of OFT septation in zebrafish, it was unclear whether there would be any CNC contribution to the zebrafish heart. However, using three lineage-tracing techniques, it has been demonstrated that they contribute to the pharyngeal arches and OFT, but additionally they were found to incorporate into the myocardium and differentiate into muscle[131]. Given the myocardial contribution, laser ablation of zebrafish CNC more severely affects ventricular function when compared with chick/mouse[132]. It is thought that perhaps birds and mammals have acquired signals that stop the CNC in the OFT, rather than traveling deeper in the heart, so they are in the right location to participate in septation. Evolutionarily, zebrafish heart development occurs without the subsequent modifications that CNC contribute to in birds and mammals, and the advancement of divided circulation could be the reason CNC switched from a myocardial to mesenchymal phenotype[131]. The CNC lineage has also been shown to be important for Xenopus heart development[98]. In Xenopus, NC migrate from the neural folds throughout the embryo and give rise to multiple cell types, but rather than the highly restricted closely related sheet of NC observed in mouse/chick embryos, the NC in Xenopus were much fewer, migrated individually, and were less restricted. When premigratory CNC were ablated in Xenopus, expression of a presumptive NC marker (xId2 transcription factor) was reduced in pharyngeal arches and absent in the OFT, inflow tract, and myocardium[98].

## CNC STEM CELLS

At the onset of migration, the NC represent a heterogeneous population of cells with regard to their developmental potentials. It has been suggested that they consist of a mixture of stem cells, fate-restricted cells, and cells that are committed to the smooth muscle cell lineage[133]. The existence of pluripotent progenitors was shown by *in vitro* clonal analysis[134,135] and by labeling individual NC cells *in vivo*[136]. Both approaches demonstrated that an individual cell can give rise to an array of differentiated progeny, including sympathetic neurons, sensory neurons, Schwann cells, melanocytes, smooth muscle cells, chondrocytes, fibroblasts, and possibly other cell types[137].

Two recent mouse *Cre/loxP* NC lineage mapping studies have similarly suggested some CNC exist in the embryonic heart that are not fully differentiated and may be multipotent[108,109]. Using the NC-restricted *P0-Cre* lineage marker mice crossed to floxed EGFP indicator line, it was shown that a small population of EGFP positive CNC in the heart colocalize with known stem cell markers[108]. Multilineage progenitor (side populations) cells in the heart were identified by cell surface marker expression, nestin, size, proliferation, ability to form spheres in culture with no serum, and the ability to lose multipotency following addition of serum. These *P0-Cre* marked cells could be expanded in culture and differentiated into cardiomyocytes, smooth muscle cells, neurons, and glia[109]. A second group also used the *P0-Cre* (and *Wnt1-Cre*) lineage marker mice, but this time crossed them to the *R26R lacZ* indicator line and found that a significant number of NC was seen to contribute to the adult semilunar and atrioventricular valves. Marked CNC were observed to enter the atrioventricular valves from both the venous and arterial poles, and were shown to contribute to the proximal conduction system at late developmental stages[108]. Intriguingly, lineage-specific antibody immunohistochemistry indicated that some NC-derived cells in the atrioventricular valves expressed melanocyte and neurogenic markers, some NC-derived in the cardiac conduction system expressed neurogenic and gliagenic markers, and another population of NC-derived cells expressed no differentiation specific markers at all[108]. Thus, it has been suggested that multipotent/stem cells with NC origin exist dormant in the neonatal heart and, on receiving the right signals, could differentiate into various cell types, thereby offering therapeutic potential.

## CONCLUSIONS

The critical requirement of the CNC during cardiovascular development is well documented, as are the severe and diverse congenital consequences associated with their removal and/or genetic manipulation (see Table 1). Both animal models and human candidate gene-mapping approaches reveal that multiple interacting signaling pathways play a role during CNC formation, migration (schematized in Fig. 5), and colonization of the heart. Local cell-cell interactions among the endothelium, AHF, pharyngeal endoderm and ectoderm, and cardiomyocytes appear critical during subsequent colonization and septation of the OFT. The challenge now lies in integrating these data and identifying the common underlying mechanisms and relationships between these implicated signaling pathways. Systemic and conditional gene deletion and lineage mapping studies have proved tremendously effective, and enabled us to build a mechanistic framework that explains some of the molecular mechanisms controlling the coupled abnormal CNC morphogenesis and myocardial dysfunction during embryogenesis. An inductive interaction between CNC and the epicardium is an attractive concept for integrating the various structural and functional defects, but presently there is only limited experimental support[122]. Similarly, a greater understanding of the recent proposed dynamic intracellular (possibly reciprocal) signaling between the CNC-AHF lineages is likely to be important for complete insight into the actual functional mechanism of the CNC within the heart. Reliable and restricted epicardial Cre-expressing mice, in conjunction with AHF-restricted Cre mice, in various combinations may be required to determine whether alteration of epicardial and/or AHF gene expression and function can be affected by CNC defects. Subsequent myocardial defects caused by a lack of appropriate epicardial and/or AHF signals might then account for ventricular dysfunction. With the likely future identification of CNC-restricted and postmigratory CNC markers, subsequent tissue-restricted and inducible targeting approaches will help to further discriminate the primary from the secondary nonspecific cardiovascular effects. The identification of several unexpected derivatives of the CNC and the possibility that pluripotent CNC reside within the heart chambers all require ongoing investigation.

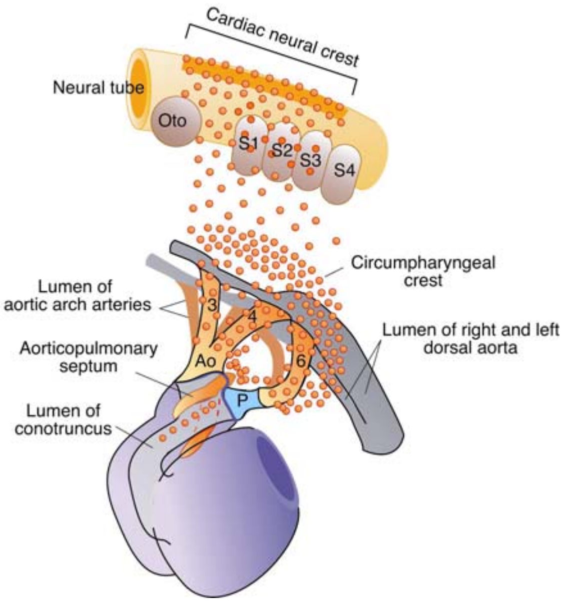

**FIGURE 5.** Schematic representation of migration of the CNC to the circumpharyngeal ridge, caudal pharyngeal arches (third, fourth, and sixth), and OFT prior to asymmetrical remodeling of the aortic arch arteries. Note that some of the CNC migrate in and surround the nascent aortic arch arteries, while others continue to migrate and eventually colonize the aorticopulmonary septum. Abbreviations: S1, S2, S3, S4, somites 1–4; Oto, otic vesicle. (From Kirby[170], with permission.)

**TABLE 1**
**Critical requirement of Cardiac NC**

| Putative CNC Role | Genes | Loss and Gain-of-Function Cardiovascular Effects | Ref. |
|---|---|---|---|
| Specification | *Pax3* | Homozygous embryos have PTA and VSD and die ~E14 due to myocardial dysfunction. | 23,24 |
| | *Wnt1/Wnt3a* | Double nulls fail to undergo NC expansion and have CNC cells stuck in the NT and die ~E18.5. | 34 |
| | *P0* | Useful marker of NC starting ~E9.0. Knockout mice are viable, but have abnormal NC-derived Schwann cells. | 17,138 |
| | *Ap2α* | Expressed in cardiac and cranial NC. Null mice die perinatally and exhibit defects in the OFT, NT, craniofacial skeleton, eye, and cranial ganglia. | 139 |
| | *Msx2* | Knockout mice are viable, but have defects in skull ossification, calvarial bones, teeth, and mammary glands. *Pax3/Msx2* double nulls rescue *Pax3* mutant OFT defects, but still have defects in muscles and NT. | 37,140 |
| | *Msx1/Msx2* | Mice homozygous for mutations in both genes die ~E18.0 with craniofacial malformations, dysmorphogenesis of pharyngeal derivatives, and anomalies in the conotruncal structures of the heart. | 141 |
| EMT | *Snail (Snai1)* | Homozygous mutants die ~E8.0 with defects in gastrulation and lack mesoderm. Ectopic expression in the chick hindbrain increases NC cell production. | 142,143,144 |
| | *Slug (Snai2)* | Knockout mice are viable, although they exhibit postnatal growth deficiency. Conversely, incubation with antisense oligonucleotides in chick embryos results in failure of presumptive NC to transform into mesenchyme. | 145,146,147 |
| | *Id2* | Mutant mice are *in utero* viable, however, ectopic expression of *Id2* in chick embryos results in conversion of ectodermal cells to NC cells. | 148,149,150 |
| | *Pinch-1* | Mull mice die ~E6.5 and overexpression in chick neural fold explants halts NC cell migration. | 41,151 |
| | *RhoB* | Chick neural tube explants treated with C3 exotoxin inhibit *RhoB* activity and prevent NC delamination. | 43 |
| | *Foxd3* | Null mice die ~E6.0 with an expansion of the extraembryonic ectoderm and loss of pluripotent epiblast. Ectopic expression in chick neural tubes induces NC marker expression, promotes delamination and NC migration. | 152,153 |
| | *Sox9* | Null embryos die ~E11.5 from congestive heart failure due to dilated major blood vessels. Null embryos exhibit hypoplasia of the branchial arches. Conditional Wnt1-Cre deletion results in NC apoptosis just before or just after migration into the periphery. | 44,154 |
| Migration | *Cx43α1* | Knockouts have enlargement of the right ventricle, attenuation of ductus arteriosus, abnormal myocardial development in the conotruncus, and die ~birth due to pulmonary OFT obstruction. | 47 |
| | *N-cadherin* | Homozygous mutant mice die ~E10.0. Conditional Wnt1-Cre deletion results in lethality ~E12.5–13.5 and the mutant mice have OFT defects, including PTA and thin ventricular myocardium with a detached epicardium. | 155,156 |

**TABLE 1 (continued)**

| Putative CNC Role | Genes | Loss and Gain-of-Function Cardiovascular Effects | Ref. |
|---|---|---|---|
| | *Wnt11* | Knockouts die ~2 days postpartum most likely due to cardiac defects. Following injection of the dominant negative form into dorsal blastomeres of *Xenopus* embryos, there is inhibition in the migration of NC. | 52,157 |
| Aortic arch remodeling | *Pdgf receptor α* | Most null mice die ~E16.5 due to extensive hemorrhaging. Conditional Wnt1-Cre studies results in neonatal lethality with cleft palate, aortic arch artery defects, VSD, and OFT defects, including PTA. | 65,158 |
| | *Alk2* | Mutant mice die ~E9.5 and severe disruption of mesoderm formation. Conditional Wnt1-Cre analysis revealed that mutants have deficiencies in NC cell migration to the OFT, lack of NC-derived smooth muscle around the aortic arch arteries with abnormal regression of the third and sixth aortic arch arteries, and PTA. | 66,159,160 |
| | *Alk5* | Deletion of Alk5 in NC cells with Wnt1-Cre causes death in mutant mice perinatally. PTA, inappropriate arch artery remodeling and aortic sac development, and abnormal thymus and parathyroids are present. | 67 |
| Arterial tree remodeling | *Foxc1/Foxc2* | Double null mutants die ~E9.5, lack both an OFT and right ventricle, exhibit NC apoptosis resulting in a spectrum of NC-related defects. | 161 |
| | *Hand1* | Homozygous embryos die ~E8.5–9.5 and exhibit yolk sac abnormalities as a result of mesoderm deficiency and undergo abnormal heart looping. | 162 |
| | *Hand2* | Null mutants die ~E10.5 due to cardiac failure, lack of aortic arch vessels, absent right ventricle, thin myocardium, and an absence of trabeculation. | 163 |
| | *Vegf-A* | Mice lacking *Vegf-A* die prior to postnatal day 14 and have enlarged hearts with irregular heartbeat and weak contractions, impaired myocardial angiogenesis, aortic arch artery defects, and fatal ischemic cardiomyopathy. | 164 |
| | *Neuropilin-1* | Most null mice die ~E13.5 and have transposition of the aortic arch arteries, PTA, and disorganized yolk sac. | 165 |
| | *Mef2c* | Homozygous mutants die ~E9.5 and have a lack of smooth muscle cell differentiation, absent right ventricle, and failure of vascular remodeling in the yolk sac. | 80,166 |
| | *Tissue factor* | Null mutants die ~E9.5–10.5, exhibit an abnormal yolk sac circulation, and deficiency of smooth muscle cells in vitelline vessels. | 81 |
| | *Endothelin-1* | Homozygous mutants have aortic arch artery and OFT defects, VSD, and enlarged right ventricle. | 83 |
| | *Ece-1* | Knockouts have cardiac OFT defects, perimembranous VSD, abnormal remodeling/regression of great vessels, and lack enteric neurons and epidermal melanocytes. | 53 |
| | *EtA* | Mutants are cyanotic due to a structural defect in the upper airway and die shortly after birth. Mutants have defects in aortic arch artery alignment, OFT development, and craniofacial structures. | 84 |
| | *Semaphorin3c* | Mutants die shortly after birth and exhibit PTA and interruption of the aortic arch. NC migration into the proximal OFT is impaired. | 85 |

**TABLE 1 (continued)**

| Putative CNC Role | Genes | Loss and Gain-of-Function Cardiovascular Effects | Ref. |
|---|---|---|---|
| | *Tbx1* | Homozygous nulls exhibit hypoplasia of the pharynx, abnormal and ectopic NC migration within the aortic arch arteries resulting in anomalous pharyngeal arch artery formation, VSD, and lack OFT septation. | 57 |
| | *Fgf8* | Hypermorphic mutants survive till term, but have small/absent thymus, craniofacial abnormalities, and malformations of both the aortic arch arteries and OFT. | 87 |
| | *Pitx2* | Homozygous mutants die ~E14.0–15.0, and have a failure of the ventral body closure, right pulmonary isomerism, swelling of the atrioventricular canals, tricuspid and mitral valve defects, and double outlet right ventricle. | 167 |
| Myocardial development | *Lbx1* | Homozygous nulls defective heart looping and increased proliferation resulting in myocardial hyperplasia. | 117 |
| | *p57Kip2* | Mutants have umbilical abnormalities, defects in position of body wall muscles, cleft palate, gastrointestinal defects, and die just after birth. This is a useful marker of ventricular trabecular development. | 168,169 |

## ACKNOWLEDGMENTS

The *Wnt1-Cre* mice were generously provided by Henry Sucov. We are grateful to the TheScientificWorld editorial team for their invitation to write this review and useful suggestions. While we have tried to review as much of the current cardiovascular NC development literature as comprehensively as possible, we regret that our review cannot include all of the field's many exciting findings. Our studies are supported, in part, by NIH T32 HL079995 postdoctoral support to PS; NIH grants HL061677 to ABF and HL60714 to SJC; and Riley Children's Foundation, Lilly Endowment, and IU Department of Pediatrics/Cardiology support for ABF and SJC.
